# Identification of Anti-Long Chain Saturated Fatty Acid IgG Antibodies in Serum of Patients with Type 2 Diabetes

**DOI:** 10.1155/2015/196297

**Published:** 2015-11-08

**Authors:** Dequina A. Nicholas, Lorena M. Salto, Ava M. Boston, Nan Sun Kim, Marco Larios, W. Lawrence Beeson, Anthony F. Firek, Carlos A. Casiano, William H. R. Langridge, Zaida Cordero-MacIntyre, Marino De Leon

**Affiliations:** ^1^Center for Health Disparities and Molecular Medicine, Loma Linda University School of Medicine, 11085 Campus Street, Loma Linda, CA 92350, USA; ^2^Division of Biochemistry, Department of Basic Sciences, Loma Linda University School Medicine, 11085 Campus Street, Loma Linda, CA 92350, USA; ^3^Center for Nutrition, Healthy Lifestyle and Disease Prevention, School of Public Health, Loma Linda University, Sanitarium Drive, Loma Linda, CA 92354, USA; ^4^Section of Endocrinology, JL Pettis Memorial VA Medical Center, 11201 Benton Street, Loma Linda, CA 92357, USA; ^5^Division of Microbiology and Molecular Genetics, Department of Basic Sciences, Loma Linda University School Medicine, 11245 Anderson Street, Loma Linda, CA 92354, USA; ^6^Whittier College, 13406 Philadelphia Street, Whittier, CA 90601, USA; ^7^Department of Physiology and Pharmacology, Loma Linda University School Medicine, 11021 Campus Street, Loma Linda, CA 92350, USA

## Abstract

High levels of serum long chain saturated fatty acids (LCSFAs) have been associated with inflammation in type 2 diabetes. Dietary SFAs can promote inflammation, the secretion of IgG antibodies, and secretion of the proinflammatory cytokine IL-1*β*. This study characterizes anti-LCSFA IgG antibodies from patients with type 2 diabetes. Serum samples from several cohorts with type 2 diabetes were analyzed for the presence of anti-LCSFA IgG, the cytokine IL-1*β*, and nonesterified fatty acids. Anti-LCSFA IgG was isolated from patient samples and used for *in vitro* characterization of avidity and specificity. A cohort participating in *En Balance*, a diabetes health education program that improved diabetes management, tested positive for anti-LCSFA IgG. Following the 3-month program, the cohort showed a significant reduction in anti-LCSFA IgG levels. Anti-LCSFA antibodies isolated from these patients demonstrated high avidity, were specific for long chain SFAs, and correlated with serum fatty acids in patients with managed type 2 diabetes. Interestingly, anti-LCSFA IgG neutralized PA-induced IL-1*β* secretion by dendritic cells. Our data shows that nonesterified SFAs are recognized by IgG antibodies present in human blood. The identification of anti-LCSFA IgG antibodies in human sera establishes a basis for further exploration of lipid induced immune responses in diabetic patients.

## 1. Introduction

Growing evidence in the literature supports the role of increased dietary intake of saturated fats in the initiation of inflammation [1, 2]. Although elevated plasma levels of nonesterified FAs have been extensively correlated to insulin resistance, the role of specific FAs, such as palmitic acid (PA), is not well understood [3, 4]. Obesity is closely associated with increased levels of proinflammatory cytokines [5]. Visceral adipose tissue is a major site of obesity-induced inflammation, and dyslipidemia is a major factor in the recruitment of activated immune cells such as macrophages, T cells, NK cells, dendritic cells, and B cells to visceral adipose tissue. Infiltrating adipose immune cells are a major source of proinflammatory cytokines in obesity-induced inflammation and type 2 diabetes [5–7]. In particular, the proinflammatory cytokine IL-1*β* can directly cause insulin resistance in insulin-sensitive cells [5, 8–11]. Moreover, PA has been shown to activate Toll-like receptor 4 on immune cells and induce secretion of IL-1*β* [12].

Recently, B cells have been recognized as a major contributor to obesity-induced inflammation [5, 13–15]. B cells are recruited to adipose tissue in response to a high fat diet [16, 17]. The importance of IgG antibodies secreted by B cells has been established in a mouse model of type 2 diabetes. For example, depletion of B cells results in protection against diabetes in mice fed with a high fat diet [18]. In addition, the transfer of IgG antibodies from obesity induced-diabetic mice to nondiabetic mice rapidly induces insulin resistance and glucose intolerance [18]. These findings suggest that B cell secretion of antibodies may be critical regulators of insulin resistance. Parallel to mice studies, humans with type 2 diabetes have disease-associated changes in B cell function, but the role of these changes in disease pathogenesis is not well established. Insulin resistance in obese individuals is linked to antibodies directed against intracellular protein antigens such as Golgi snap receptor complex 1 and Bruton's tyrosine kinase [18]. There is the possibility that antibodies to lipids are generated in response to a high fat diet because the authors of that study only screen serum for protein antigens (B cells promote insulin resistance through modulation of T cells and production of pathogenic IgG antibodies). For instance, antibodies to cholesterol have been detected in human serum [19]. Moreover, IgM antibodies against FAs have been reported in multiple sclerosis as well as in human immunodeficiency virus (HIV) patients [20–22]. However, there is a gap in the literature of studies demonstrating the presence of IgG antibodies against FAs such as palmitic acid.

The purpose of the present study was to investigate whether humans produce class switched IgG antibodies that recognize saturated FAs such as PA. To answer this question, we retrospectively analyzed serum from 2 different cohorts of obese individuals, including patients with and without type 2 diabetes and patients who participated in the diabetes intervention program,* En Balance* [23].

## 2. Materials and Methods

### 2.1. Research Design and Methods

This study consisted of analysis of serum samples from the Bioserve biorepository in addition to serum samples from a 3-month diabetes education intervention (*En Balance*) designed to promote improved type 2 diabetes management in Hispanic adults [24–27]. We measured IL-1*β* and antibodies which recognize palmitic acid in these samples and correlated the values obtained from the* En Balance* samples with the original primary outcomes of that study. These outcomes included fasting blood glucose, HbA1c, and body composition. A total of 73 Hispanic males and females with type 2 diabetes met the* En Balance* participation criteria as previously described [26, 27].

### 2.2. Ethics and Informed Consent (*En Balance* Study)

The Loma Linda University Institutional Review Board (IRB) approved the* En Balance* study protocol and all participants gave written informed consent to participate. Signed consent forms for the study are stored in locked filing cabinets and cannot be linked to participant data according to Loma Linda University IRB protocol.

### 2.3. Evaluative Measures (*En Balance* Study)

#### 2.3.1. Glucose, A1C, and Insulin

Two blood samples (12–14 hr fasting) were drawn from the participants at both baseline and 3 months and analyzed for glucose, A1C, and insulin. Additional samples were stored frozen at −80°C for future analysis.

#### 2.3.2. Anthropometric Measures and Body Fat Composition

Anthropometric measures (height, weight, waist circumference, hip circumference, and waist/hip ratio) were assessed at baseline and 3 months as previously described [25, 28]. Body composition was assessed at baseline and at 3 months using a TANITA scale (Detecto, Web City, Missouri), bioelectric impendence technology, and a fan beam dual X-ray absorptiometry (DXA) Hologic Discovery A software version 12.6 (Waltham, MA) as previously described [26, 27].

### 2.4. Serum Samples for Detection of Anti-LCSFA IgG and IL-1*β*

Serum samples from 46* En Balance* participants were available for anti-LCSFA antibody and IL-1*β* testing. The baseline characteristics of these participants are presented in Table 1 with the exception of missing participant data. Twenty-one of these participants had paired serum samples from baseline and 3 months available for longitudinal analysis. Twelve participants had serum samples from only baseline and 13 participants had serum samples from only 3 months.

**Figure 1:**
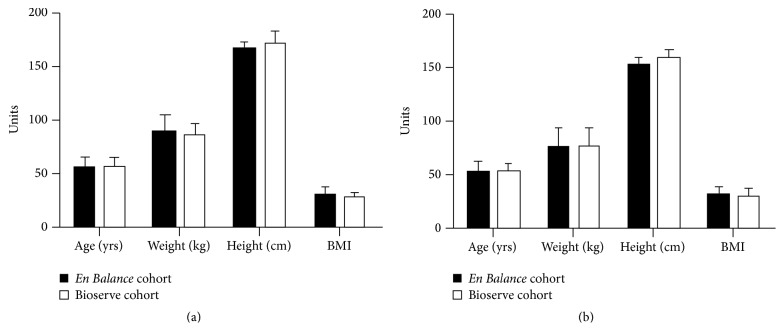
Basic patient characteristics between the* En Balance* and Bioserve cohorts do not differ. (a) Comparison of patient characteristics of male serum donors. Bar graph includes data from all* En Balance* samples (including the 21 samples for which the Bioserve samples were matched) and Bioserve samples. (b) Comparison of patient characteristics of female serum donors. Bar graph includes data from all* En Balance* samples (including the 21 samples for which the Bioserve samples were matched) and Bioserve samples.

Serum samples (four groups with 20 serum samples each: Hispanic control, Hispanics with type 2 diabetes, Caucasian control, and Caucasians with type 2 diabetes) purchased from the Bioserve biorepository (Beltsville, MD) were also tested for anti-LCSFA antibody and IL-1*β*. The samples were gender, age, and BMI matched against the 21* En Balance* participants with paired samples (Table 2 and Figure 1). Exclusion criteria consisted of history of renal disease, self-declared diabetic complications, tobacco use, and other medical histories including cancer history.

### 2.5. Synthesis of Palmitoylated Bovine Serum Albumin

Palmitoylation of BSA was performed by modifying a protocol developed by the Geffard laboratory [20–22]. For this reaction, 10 mg of PA was dissolved in 10 mL of anhydrous methanol (Sigma-Aldrich) containing 100 *μ*L of triethylamine (Sigma-Aldrich). To activate the carboxylic acid group, 200 mL of anhydrous dimethylformamide (DMF) solution containing ethylchloroformate (ETOCOCL) (Sigma-Aldrich) diluted 1/16 was added. The solution was incubated for 3 min at 4°C. Next, 200 mg FA-free BSA (Gemini Bio-Products) was dissolved in 10 mL of 1 M phosphate buffer (pH 6.8), containing 1 mM CaCl_2_ and 100 *μ*L of triethylamine (Sigma-Aldrich). The BSA solution was added to the PA solution and the mixture was stirred for 1 hr at room temperature. The BSA-PA conjugates were purified by dialysis against 150 mL of 1 mM CaCl_2_ phosphate buffer (pH 6.8), containing dimethylformamide and methanol, (vol/vol/vol) overnight at room temperature (RT). The conjugate was then dialyzed against 1 M phosphate buffer (pH 6.8), containing 1 mM CaCl_2_ for 24 hours at RT. The mixture was then dialyzed against PBS (pH 7.2) until precipitated phosphate became soluble. The BSA-PA was filtered to remove any remaining precipitate and the BSA-PA concentration was determined by measuring the optical density (OD) at 280 nm.

### 2.6. ELISA

An ELISA was developed for identifying IgG antibodies in human serum that recognize PA using the Ready-Set-Go ELISA IgG kit (eBioscience) according to manufacturer's protocol with the following exceptions. Plates were coated with 100 *μ*L of 50 *μ*g/mL PA-BSA or BSA. Serum samples were diluted 1 : 100 before use in ELISA. All samples were analyzed in triplicate.

Antibody avidity was determined by a competition ELISA as previously described [22]. Purified IgG at 2 *μ*g/mL in PBS was preincubated for 16 hrs at 4°C with BSA-PA at concentrations varying between 10^−4^ and 10^−11^ M. After centrifugation at 10,000 ×g for 30 min, 100 *μ*L of the supernatants was analyzed by ELISA as described above. Antibody avidity was defined as the concentration of BSA-PA required for 50% inhibition (IC_50_) of antibody binding to immobilized BSA-PA.

### 2.7. Isolation of Anti-LCSFA IgG Antibodies

Total IgG from 7 diabetic patient participant sera was isolated and purified on Pierce Protein G Chromatography Cartridges (Thermo Scientific) according to the manufacturer's protocol. IgG that recognized BSA-PA was purified using the SulfoLink Immobilization Kit for Proteins (Thermo Scientific) according to manufacturer's instructions. BSA-PA was immobilized to resin via covalent thioester bonds, and this resin was used to pack the chromatography column. The anti-LCSFA antibodies (antibodies isolated by affinity to BSA-PA) were then purified from their respective IgG fraction by affinity chromatography according to the protocol outlined for the SulfoLink Immobilization Kit (Thermo Scientific).

### 2.8. Determination of Anti-LCSFA IgG Specificity with Lipid Dot Blot

A lipid dot blot method was established to determine the reactivity of a panel of nonesterified FAs (from Sigma-Aldrich) and esterified palmitic acid (glycerol tripalmitate from Sigma-Aldrich and N-palmitoyl phosphatidyl ethanolamine from Enzo Life Sciences) to purified anti-LCSFA IgG. Two *μ*L of 0.2 M FAs dissolved in 100% chloroform + 0.1% HCL was spotted onto nitrocellulose. After allowing the samples to completely dry, the membrane was blocked in Odyssey blocking buffer for 2 hrs. The lipid blots were probed overnight at 4°C with purified antibodies (diluted 1 : 1000) that recognize BSA-PA. After washing the blot with PBS, anti-Human IgG alkaline phosphatase conjugate (Sigma) was used as the secondary (1 : 10,000) detection Ab.

### 2.9. Anti-LCSFA IgG Neutralization Assay

Monocytes (CD14^+^) were isolated from human peripheral blood (Lifestream blood bank) and cultured at 37°C and 5% CO_2_ in 96-well plates at a density of 200,000 per well in RPMI-1640 supplemented with 10% FBS, 1,000 U penicillin and streptomycin, 50 ng/mL GM-CSF, and 10 ng/mL IL-4 for 6 days to differentiate them into dendritic cells (DCs). The DCs were treated with 150 *μ*M PA in a 1 : 1 ratio with BSA for 24 hrs in the presence of IgG Ab containing anti-LCSFA IgG. A mixture of total IgG isolated from 5 Hispanics with type 2 diabetes with high anti-LCSFA IgG levels (measured by ELISA) or a mixture of total IgG isolated from 2 samples which tested negative for anti-LCSFA IgG (measured by ELISA) was used at a concentration of 1.4 mg/mL. Preabsorption of PA was performed at 37°C for 2 hrs. Fifty *μ*L of culture supernatant was analyzed by cytometric bead array.

### 2.10. Determination of Serum Nonesterified Fatty Acid Concentration

Using the Free Fatty Assay Colorimetric Kit (Cell Biolabs, Inc.), the concentration of free fatty acid in the serum samples diluted 1 : 2 was determined in duplicate following the manufacturer's recommendations.

### 2.11. Cytometric Bead Array

*En Balance* serum samples and cell culture supernatants were analyzed with a human IL-1*β* flex set as described by the manufacturer (BD Biosciences) on a MacsQuant flow cytometer (Miltenyi Biotech). Data analysis was accomplished with FACP array v3 software.

### 2.12. Western Blot

**Figure 2:**
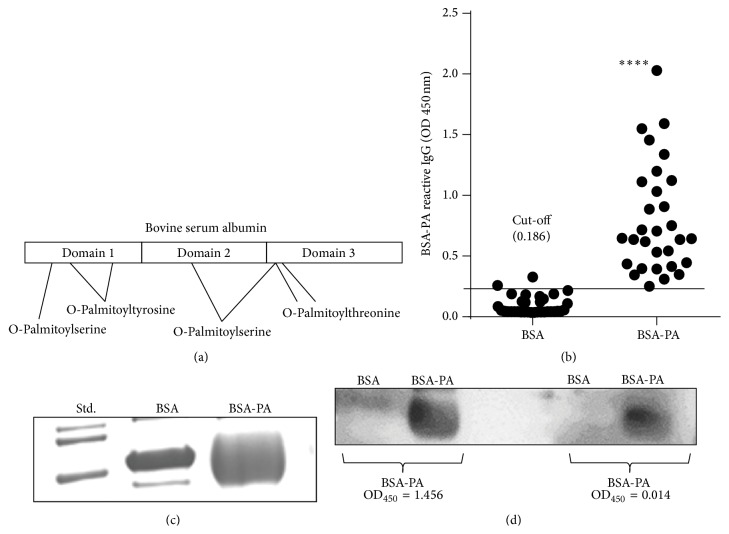
Patient serum reacts with palmitoylated BSA. (a) Diagram of BSA palmitoylation sites. Seven accessible hydroxyl groups that can be palmitoylated are present on serine, tyrosine, or threonine of BSA. (b) ELISA identification of BSA-PA reactive IgG antibodies.* En Balance* baseline serum samples from Hispanic diabetic patients were tested by ELISA for IgG Abs that react with BSA (2/36) or BSA-PA (36/36). The cut-off value for positive antibody reactivity against BSA-PA (>0.186) was defined as an absorbance greater than two standard deviations above the mean value for BSA. (c) A Coomassie stained gel of palmitoylated BSA (BSA-PA). Palmitoylated BSA migrates faster on the gel than BSA due to an increased (−) charge contributed by SDS association with the palmitoyl groups. (d) Western blot can detect differential levels of Ab that react with BSA-PA. Western blots with BSA alone (10 *μ*g) and BSA-PA (60 *μ*g) were probed with total serum from Hispanics with type 2 diabetes which had a high OD_450_ (OD = 1.456) or low OD_450_ (OD = 0.014) as determined by ELISA for reactivity with BSA-PA. Anti-human IgG was the secondary antibody. (c) Statistical analysis was accomplished with a Wilcoxon-ranked sign test. ^*∗∗∗∗*^ *p* < 0.0001.

Western blots were performed on ~10 *μ*g BSA or 60 *μ*g BSA-PA. Due to the diffuse banding pattern of BSA-PA, increased amounts of BSA-PA (60 *μ*g) were loaded for the western blot (Figure 2(c)) in order to detect differences between samples. The proteins were separated on a 12% acrylamide gel at 120 V and then transferred to PVDF membranes and blocked with 5% nonfat milk solution in Dulbecco's PBS (Caisson Labs, Logan, UT) for 1 hr at room temperature. After blocking, the membrane was incubated in primary antibody (complete patient serum) diluted 1 : 100 in 5% nonfat milk solution overnight at 4°C. The membrane was washed three times with PBS + 0.02% Tween 20 and incubated with anti-human IgG conjugated to alkaline phosphatase (Sigma) diluted 1 : 5,000 for 1 hr at room temperature. The membrane was washed three times with PBS + 0.02% Tween 20 and then one time with PBS and the protein bands were visualized on photographic film (Kodak) using Novex AP Chemiluminescent Substrate (Invitrogen).

### 2.13. Statistical Analysis

Statistical analyses were calculated using SPSS for Windows Version 22 (SPSS, Inc., Chicago, Illinois) with type I error set at *α* = 0.05. The data were presented as the means ± SD. Univariate and bivariate analyses were reported at baseline and at 3 months. Spearman's correlation coefficient was used to determine associations between body composition, glucose, and insulin levels with Il-1*β* and anti-LCSFA antibodies. The Wilcoxon signed-rank test was used to identify baseline to 3-month paired differences. Mann-Whitney test was used for nonparametric unpaired data analysis of changes in anti-LCSFA antibody and FFA levels. GraphPad Prism 6 nonlinear regression with outliers automatically excluded was used to determine IC_50_.

## 3. Results

### 3.1. Detection of Anti-LCSFA IgG Antibodies by ELISA

**Figure 3:**
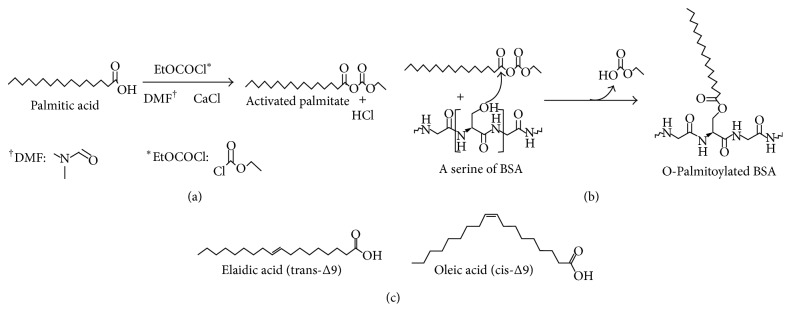
O-Palmitoylation of BSA. (a) The donated hydrogen from PA associates with Cl^−^ to form HCl and two palmitate anions associate with Ca^2+^ to form calcium palmitate. The negatively charged palmitate allows a nucleophilic attack on ethylchloroformate (EtOCOCL). The Cl^−^ is removed as a leaving group and an activated palmitate (anhydride) is formed. DMF: dimethylformamide. (b) Available hydroxyl groups on BSA perform a nucleophilic attack on the activated palmitate via an SN2 mechanism. The leaving group is removed and the BSA is O-palmitoylated. (c) Chemical structures of elaidic acid and oleic acid.

Lipids are generally considered to be poor immunogens, and immunoassays targeting lipids like PA are rare. We detected IgM (data not shown) and IgG antibodies which recognize BSA-PA in serum by using a specialized ELISA (Figure 2) [21]. Because adsorbent 96-well plates bind protein and not FAs, we conjugated the FA of interest, PA, to BSA (BSA-PA) in order to stably coat the plates with PA (Figures 2(a) and 2(c)). The acylation reaction of BSA with PA occurs via an SN2 mechanism which results in O-palmitoylation of serine, tyrosine, and threonine in BSA (Figures 2(a), 3(a), and 3(b)). Due to the orientation of the palmitoyl groups on BSA, only the carbon chain is an accessible epitope for Ab binding (Figure 3(b)). We adopted this published approach with slight modifications and demonstrated that class switched IgG antibodies which recognize PA can be identified in human serum (Figure 2(b)) [22]. Additionally, we tested whether serum samples with differential reactivity for BSA-PA according to ELISA could also be distinguished by western blot (Figure 2(d)). The serum with higher OD_450_ did exhibit stronger reactivity with BSA-PA than sera with a lower OD_450_.

### 3.2. Anti-LCSFA IgG Antibody Specificity for Long Chain Saturated FAs

**Figure 4:**
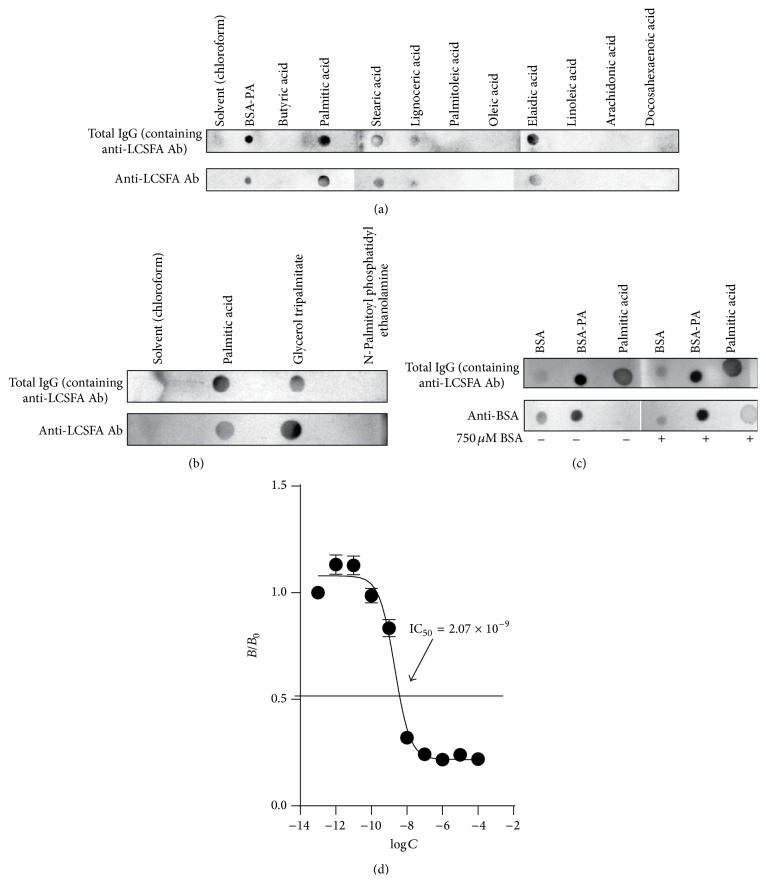
Specificity of diabetic patient anti-LCSFA IgG antibodies for long chain saturated FAs. (a) Lipid dot blot of a panel of nonesterified FAs. Dot blots presented are from one patient serum and are representative of dot blots performed with antibodies isolated from the sera of 5 diabetic patients. The FAs were probed with purified total IgG or purified anti-LCSFA IgG isolated from the same individual. Anti-human IgG conjugated to alkaline phosphatase was used as the secondary Ab. The blots are representative of separate experiments performed with Abs isolated from the sera of 1 of 5* En Balance* participants positive for anti-LCSFA IgG. (b) Lipid dot blot comparison of nonesterified and esterified palmitic acid. The palmitic acid molecules were probed with purified IgG or purified anti-LCSFA IgG as the primary Ab. Anti-human IgG conjugated to alkaline phosphatase was the secondary Ab. The dot blots show an experiment from one individual representative of 3 experiments performed with Abs isolated from 3* En Balance* participants. (c) Dot blot binding assay from a representative patient demonstrating anti-LCSFA binding palmitic acid in the presence of physiological levels of BSA (750 *μ*M). (d) Competition ELISA determination of anti-LCSFA IgG avidity. log⁡*C* is the log_10_⁡  of the concentration of the competitor BSA-PA in mol/L. The ratio between the absorbance with competition (*B*) and without competition (*B*_0_) is plotted. Each data point represents the mean ratio of the absorbance ± SEM of antibodies from 5 participant sera.

To further characterize the antibodies which recognize BSA-PA, we isolated the total IgG fraction and from this IgG fraction we also isolated IgG antibodies which recognize BSA-PA from 5* En Balance* participants with the highest antibody levels to determine Ab specificity and avidity. Using a novel lipid dot blot method and a panel of fatty acids (Table 3), we found that these antibodies can recognize nonesterified palmitic acid (16 : 0). In addition, we found that the antibodies cross-react with stearic acid (18 : 0), lignoceric acid (24 : 0), and elaidic acid (19 : 1) (Figure 4(a)). Because the BSA-PA reactive antibodies cross-react with long chain saturated fatty acids (LCSFA), we termed these antibodies anti-LCSFA IgG. We also determined that the BSA-PA reactive IgG recognizes palmitic acid esterified to glycerol but not to phosphate (Figure 4(b)). Both total IgG fractions and the purified BSA-PA reactive IgG reacted with the same fatty acids. Next, we modified this dot blot approach to determine whether the antibodies could bind to nonesterified PA in the presence of physiological concentrations of BSA.  Figure 4(c) indicates that a PA dot blot probed with total IgG fraction containing BSA-PA reactive IgG still binds to PA in the presence of 750 *μ*M BSA. In addition, we determined the avidity of the IgG antibodies for BSA-PA. Following the protocol published by Boullerne et al. [22], we used a competition ELISA to determine that the avidity of anti-LCSFA IgG to BSA-PA was approximately 2.07 × 10^−9^ (Figure 4(d)).

### 3.3. Anti-LCSFA IgG Antibodies Are Detectable in Both Diabetic and Nondiabetic Cohorts

**Figure 5:**
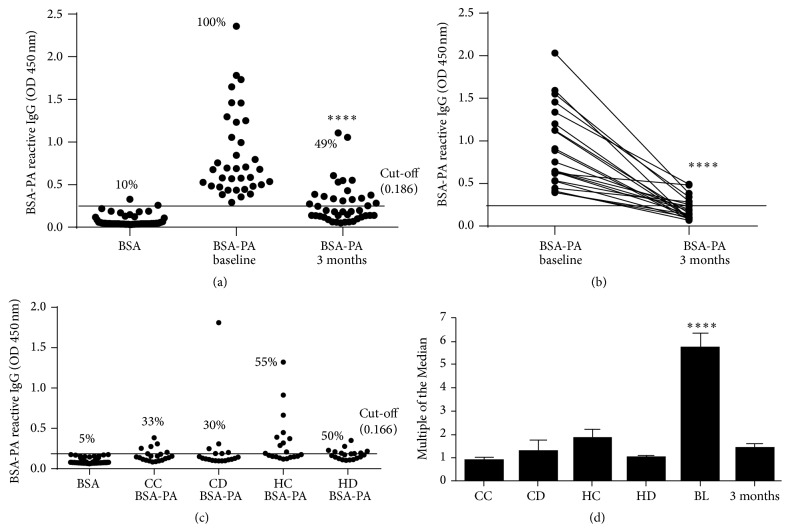
Detection of anti-LCSFA antibodies in human serum. (a) Anti-LCSFA IgG in serum of Hispanic* En Balance* participants with type 2 diabetes is reduced following 3 months of diabetes education. Each data point represents the mean optical density of anti-LCSFA IgG antibodies detectable by ELISA. The cut-off value for positive antibody reactivity against BSA-PA (>0.186) was defined as an absorbance greater than two standard deviations above the mean value for BSA. The frequency of sera positive for each group is indicated as a percent on the graph. (b) Paired data from the 21* En Balance* participants with both baseline (mean ± SEM = 1.05 ± 0.121) and 3-month serum (mean ± SEM = 0.25 ± 0.030) samples. The black lines connect data points belonging to the same participant. Data was analyzed by Mann-Whitney (for (a)) and Wilcoxon test (for (b)). (c) Frequency of anti-LCSFA IgG Abs in serum samples from Bioserve biorepository. CC: Caucasian control; CD: Caucasian diabetic; HC: Hispanic control; HD: Hispanic diabetic. The cut-off value for positive antibody reactivity against BSA-PA (>0.166) was defined as an absorbance greater than two standard deviations above the mean value for BSA. (d) Comparison of anti-LCSFA IgG levels among* En Balance* serum samples and Bioserve biorepository serum samples. The median of all corrected OD values (OD_450_ of BSA-PA reactivity − OD_450_ of BSA reactivity) for each sample was determined. The corrected OD value for each sample was divided by the median to obtain the Multiple of the Median (MoM). A MoM value of 1 is considered the “normal” range for the anti-LCSFA antibody. BL:* En Balance* baseline samples; 3 Mo:* En Balance* 3-month samples. Data was analyzed by Mann-Whitney. ^*∗∗∗∗*^ *p* < 0.0001.

After validation of the ELISA technique to identify anti-LCSFA antibodies, we set out to determine whether these antibodies play a role in type 2 diabetes. We tested for the presence of PA antibodies in the sera of 46 Hispanics with type 2 diabetes and assessed the impact of the* En Balance* program on antibody levels. Out of a total of 67 serum samples, paired samples (baseline versus 3-month intervention) were available for only 21 participants. The remaining 26 participants had only a baseline or three month serum sample. We found that 100% (36/36) of the Hispanics with type 2 diabetes tested positive for anti-LCSFA IgG antibodies before undergoing the diabetes education program (Figure 5(a)). Interestingly, after 3 months of participation in the* En Balance* program, the frequency of participants that tested positive for anti-LCSFA antibodies significantly decreased to 49% (20/41) (Figure 5(a)). More importantly, the relative levels of anti-LCSFA antibodies in individual participants (*n* = 21) from baseline (mean ± SEM = 1.05 ± 0.121) to 3 months (mean ± SEM = 0.25 ± 0.030) were significantly reduced (Figure 5(b)). This reduction in anti-LCSFA antibodies coincides with improved diabetes control as described in previous publications (Table 4) [23, 25–28].

To determine whether these results are cohort, disease, or ethnicity specific, we tested serum samples from the Bioserve biorepository. We found that approximately 30% of Caucasian control samples (7/21) and 33% of samples from Caucasians with type 2 diabetes (6/20) tested positive for anti-LCSFA IgG by ELISA (Figure 5(c)). Overall, samples from Hispanic cohorts showed an increased frequency of patients who tested positive for anti-LCSFA IgG. Sera from Hispanic controls were 55% positive (11/20) while sera from Hispanics with type 2 diabetes were 50% positive (10/20). Interestingly, anti-LCSFA antibodies levels were higher in the Bioserve Hispanic control group than in the Bioserve Hispanic diabetic cohort. The frequencies of serum samples positive for anti-LCSFA IgG in the Hispanic cohorts from the Bioserve biorepository (55% and 50%) were similar to that of serum samples from the* En Balance* (49%) cohort after 3 months of intervention.

Lastly, to compare anti-LCSFA antibody levels across all groups, we performed Multiple of the Median (MoM) analysis. This type of analysis normalizes data with “1” corresponding to “normal” levels and also measures how far an individual test result deviates from the median. MoM is commonly used to report the results of medical screening tests that tend to be highly variable [29]. First, we corrected the anti-LCSFA IgG optical density values at OD_450_ by subtracting out the OD_450_ values obtained by reacting each serum sample with BSA alone. We then found the median of the all the corrected OD_450_ values from both the Bioserve and the* En Balance* samples. Each sample's OD_450_ value was divided by this median to obtain the MoM value. We found that only the baseline serum samples from the* En Balance* study significantly deviated from “normal” values in this data set (Figure 5(d)).

### 3.4. Anti-LCSFA Antibodies and Serum IL-1*β* from Hispanic Participants with Type 2 Diabetes Correlate with Diabetes Health Variables

In addition to measuring PA antibodies, we determined the concentration of IL-1*β* present in the serum of* En Balance* participants. We found that serum IL-1*β* levels did not change from baseline to 3 months (Table 4). Next, we performed multivariate analysis of anti-LCSFA antibodies, IL-1*β*, and the health variables collected from each participant at baseline and at 3 months. Surprisingly, we found that anti-LCSFA antibodies negatively correlated with body fat (Table 5). In addition, the levels of IL-1*β* correlated to HbA1c values (Table 5).

### 3.5. Total Nonesterified Fatty Acid Is Not a Direct Indicator of Anti-LCSFA IgG Levels

**Figure 6:**
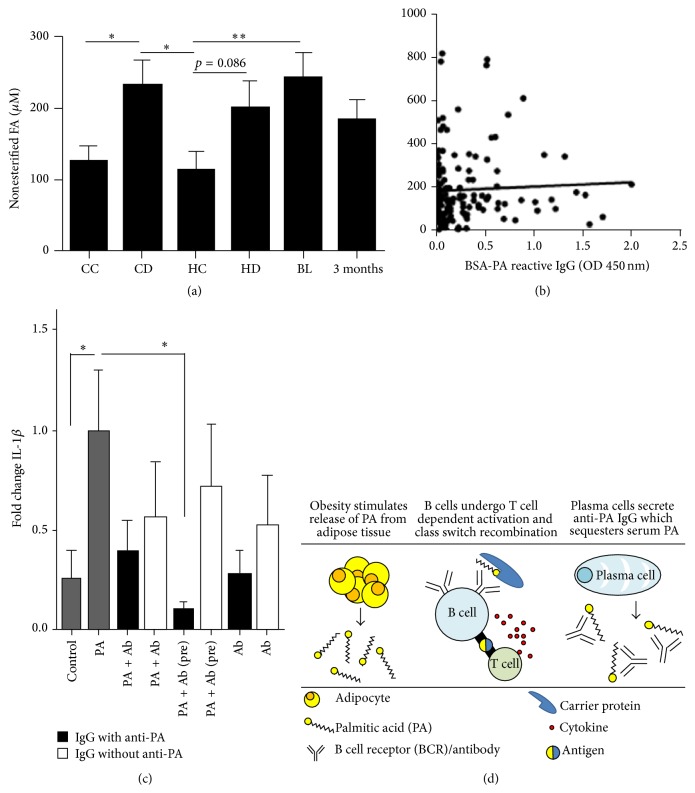
Serum nonesterified serum FA. (a) Bar graph of serum nonesterified FA among diabetic and nondiabetic cohorts. CC = Caucasian control; CD = Caucasian diabetic; HC = Hispanic control; HD: Hispanic diabetic. Data was analyzed by Mann-Whitney. ^*∗*^ *p* < 0.05, ^*∗*^ *p* < 0.01. (b) *XY* plot of anti-LCSFA IgG and nonesterified serum FA for all serum samples in this study. (c) Anti-LCSFA IgG neutralizes PA-induced IL-*β* secretion from DCs in the presence of BSA. Monocyte derived dendritic cells from healthy patients were cultured with 150 *μ*M PA (in 1 : 1 ratio with BSA) in the presence of a mixture of IgG antibody isolated from 5* En Balance* participants who tested positive for anti-LCSFA IgG (black bars) or in the presence of a mixture of IgG antibody isolated from 2* En Balance* participants who tested negative for the antibody after the 3-month intervention (white bars). PA + Ab (pre) indicates dendritic cells that were treated with PA preabsorbed for 2 hrs with total IgG from the two groups described. After 24 hrs, media from the cell cultures were analyzed by cytometric bead array for IL-1*β*. Data was analyzed by one-way ANOVA. ^*∗*^ *p* < 0.05. (d) Illustration of the potential mechanism for the generation of anti-LCSFA IgG.

We measured the concentration of total nonesterified fatty acid (both bound and free) in all serum samples. Confirming the literature, total FA in serum was significantly higher in the cohorts with type 2 diabetes as compared to the controls without diabetes (Figure 6(a)). When combining all cohorts, the levels of FA did not correlate with the levels of anti-LCSFA IgG detected in the serum samples (Figure 6(b)). Because no significant correlations of anti-LCSFA antibodies with serum FA were found overall, we subcategorized the samples and analyzed the data according to the published American Diabetes Association target ranges for fasting blood glucose (FBG). We found that the levels of FFA correlate to anti-LCSFA IgG levels (*n* = 24, Spearman's rho 0.4862, *p* = 0.16) for individuals whose FBG was in the target range of 70–130 mg/dL. In other words, the levels of FFA correlate to anti-LCSFA IgG levels for individuals whose diabetes is managed.

### 3.6. Anti-LCSFA Antibodies Neutralize PA-Induced Secretion of Dendritic Cell IL-1*β*

Finally, to further understand the functions anti-LCSFA antibodies may play in humans, we determined the effect of anti-LCSFA antibodies on PA-induced secretion of IL-1*β* from dendritic cells in the presence of BSA (Figure 6(c)). We found that preabsorption of PA with IgG antibodies from patients with high levels of anti-LCSFA IgG significantly reduced DC secretion of IL-1*β* (Figure 6(c)). However, a significant reduction in DC secretion of IL1*β* was not observed in patients who tested negative for anti-LCSFA by ELISA. This result suggests that anti-LCSFA IgG may sequester nonesterified FAs in the blood. In support of this hypothesis, we found that anti-LCSFA antibodies negatively correlate with serum IL-1*β* in the En Balance cohort at baseline (*n* = 33, Spearman's *r* = −0.34, *p* = 0.026) and at 3 months (*n* = 33, Spearman's *r* = −0.26, *p* = 0.05).

## 4. Discussion

In this paper we show that IgG antibodies against PA and other saturated fatty acids are detectable in human serum (Figures 2 and 5). The activity of IgG antibodies occurs principally during a secondary antibody response driven by T cells. Therefore, the emergence of anti-LCSFA IgG antibodies coincides with maturation of an antibody response, which occurs upon repeated exposure to an antigen. We have also demonstrated that conjugation of a fatty acid to the carrier protein BSA is unnecessary for anti-LCSFA IgG recognition of saturated fatty acids (Figure 4(a)). The anti-LCSFA antibodies we detected can recognize nonesterified palmitic acid as well as palmitic acid esterified to glycerol (Figure 4(b)), indicating that these antibodies could potentially function both in the blood and in the tissue where saturated fatty acids are stored as triglycerides.

Characterization of anti-LCSFA IgG antibodies revealed that they are not specific for PA but can also bind to other long chain saturated FAs (Figure 4 and Table 3). Anti-LCSFA IgG did not recognize butyric acid (6 : 0), which indicates that these antibodies may be unable to recognize short chain saturated FAs. Interestingly, we found that anti-LCSFA did not recognize oleic acid (18 : 1) but did recognize elaidic acid (19 : 1). Both oleic and elaidic acid are monounsaturated long chain FAs; however, only elaidic acid is recognized. We speculate that this result is due to the double bond being in the trans- rather than cis-configuration (Table 3, Figure 3(c)), leading to a configuration structurally mimicking a saturated FA (Figure 3(a)). Further, we did not detect any IgG antibodies which bind to polyunsaturated FAs, supporting the notion that polyunsaturated FAs are anti-inflammatory [30]. Overall, the results presented in Figure 3 implicate that the epitope which anti-LCSFA antibodies recognize is dependent on the carbon chain length of the fatty acid as well as its configuration. Thus, it is possible that a diet high in saturated fat may be linked to anti-LCSFA antibody generation.

Of particular significance is that our data clearly show that anti-LCSFA antibodies were reduced in serum samples obtained from Hispanics with type 2 diabetes after 3 months of culturally sensitive diabetes education (Figure 5). In this same cohort of diabetic patient participants, blood glucose, HbA1c, body fat, and dietary fat were also found to be significantly reduced, indicating that managing diabetes through diet and exercise results in a reduction of PA antibody titers (Table 4 and [23, 25–28]). Therefore, we can speculate that a mechanism indirectly related to serum FA levels or dietary intake of saturated fat may be responsible for the generation of anti-LCSFA antibodies.

Our data also showed that anti-LCSFA IgG antibodies are found in both the diabetic and nondiabetic condition (Figure 5). In the Caucasian cohort, the levels of antibody did not differ between diabetic and nondiabetic serum samples. This finding indicates that the generation of anti-LCSFA antibodies may be a natural response to FAs rather than a pathological response. The detection of BSA-PA reactive antibodies by western blot in serum samples which tested negative by ELISA further support this notion (Figure 2(b)). As a natural antibody, anti-LCSFA could potentially play a role in the clearance of saturated fatty acids from the blood stream. This hypothesis is supported by our data that show that the antibodies have the ability to sequester PA and protect DCs from a proinflammatory response (Figure 6(c)). Interestingly, Hispanic participants presented with the anti-LCSFA antibody about 20% more frequently than Caucasian subjects (Figure 5(c)). To our surprise, the serum from Bioserve Hispanic nondiabetic controls had significantly higher levels of anti-LCSFA IgG than the serum from Bioserve Hispanic diabetes samples (Figure 5(c)). This observed difference could be due to variables for which we could not control in the Bioserve samples such as fasting blood glucose, time of sample draw, dietary intake, and ethnic homogeneity. The* En Balance* serum samples were from Mexican Americans as opposed to the Hispanic Bioserve samples, which represent 17 countries of origin in addition to different levels of socioeconomic status. Previous studies have shown that socioeconomic status is associated with cell mediated immunity [31]. The Caucasian Bioserve samples were more homogeneous and represent only 6 countries of origin. Although the Bioserve Hispanic controls had higher levels of anti-LCSFA IgG than the Bioserve Hispanic diabetes group, MoM analysis indicated that both of these groups reside within the prospective “normal” range for presence of the antibody. The only group that had serum levels of anti-LCSFA IgG above control or “normal” levels were from the* En Balance* participants at baseline (Figure 5(d)). Not only did these participants have type 2 diabetes, but at baseline, their diabetes was unmanaged (Table 4). This may suggest that high anti-LCSFA antibody titers are related to how well type 2 diabetes is managed. Further characterization of the relationship between diabetes management and anti-LCSFA IgG antibody levels could potentially lead to using this antibody as an additional biological indicator in the management of type 2 diabetes.

Table 5 demonstrates that anti-LCSFA antibodies negatively correlate with body fat and that IL-1*β* positively correlates with HbA1c. These correlations seem counterintuitive because increased adiposity is associated with low-grade chronic inflammation. However, obesity and increased BMI have been associated with impaired antibody responses [32–34]. Our study shows that increased fat is associated with reduced production of anti-LCSFA antibodies, a result which corroborates the literature findings [32–34]. The correlation of serum IL-1*β* with HbA1c supports the current convention that IL-1*β* is strongly associated with insulin resistance and type 2 diabetes [5, 35].

We found that IgG from Hispanics with type 2 diabetes neutralized the secretion of IL-1*β* from DCs (Figure 6(c)). Previous studies have determined that IgG antibodies generated in type 2 diabetes are pathogenic [17, 18]. An alternative possibility that requires further exploration is that anti-LCSFA IgG antibodies are generated as a protective mechanism against increased plasma nonesterified saturated FAs and subsequent inflammation including insulin resistance (Figure 6(d)). In “managed” diabetes, total serum nonesterified FAs correlate with anti-LCSFA IgG levels. However, nonesterified FAs do not correlate in “unmanaged” diabetes, indicating dysregulation of what may be a normal process of antibody production. Further research is necessary to determine what mechanisms link saturated fatty acids to the production of anti-LCSFA IgG. Based on the available data, our current hypothesis is that increased nonesterified PA resulting from excess calories (or overeating) and obesity stimulates the production of anti-LCSFA antibodies through an indirect mechanism such as palmitoylating serum proteins (Figure 6(d)). Because we observed class switched IgG antibodies (Figures 2, 4, and 5), we speculate that generation of anti-LCSFA antibodies may be antigen and T cell dependent [36]. These antibodies may then sequester nonesterified SFAs and help mitigate SFA induced inflammatory responses. However, the natural function of anti-LCSFA IgG in the body remains unknown. If the role of anti-LCSFA IgG in the healthy state and in unmanaged type 2 diabetes can be further validated, a new avenue in understanding and treatment of these conditions is possible. Potentially, these antibodies could be used as a therapeutic providing they are protective. Alternatively, they could be used as indicators of the level of diabetes management. Understanding how poor type 2 diabetes management impacts anti-LCSFA IgG production and lipid immunology could be a major influence in the future of type 2 diabetes research.

## 5. Conclusion

The main objective of the present study was to determine whether there are detectable levels of anti-LCSFA antibodies in normal or diabetic patients and our findings support the existence of such antibodies. Future studies need to explore in depth the significance of this finding using larger cohorts of normal, prediabetic and diabetic patients. We propose that our findings raise the need to investigate the role of lipid antibodies in healthy conditions and in type 2 diabetes to further our understanding of lipid immunology.

## Figures and Tables

**Table 1:** *En Balance* study participant characteristics at baseline (*n* valid and missing cases = 46, *n* missing cases for age = 5, *n* missing cases for weight, height, and BMI = 9).

	Males	Females
Age (years)	(*n* = 8)	(*n* = 33)
25–39	—	4
40–59	5	22
60+	3	7
Mean ± SD	56.25 ± 8.94	53.94 ± 10.17
Weight (kg)	(*n* = 8)	(*n* = 27)
50–75	1	16
76–99	6	8
100+	1	3
Mean ± SD	88.98 ± 16.28	75.79 ± 18.55
Height (cm)	(*n* = 8)	(*n* = 27)
145–159	—	22
160–169	4	5
170+	4	—
Mean ± SD^*∗∗∗∗*^	168.43 ± 4.60	153.83 ± 5.78
BMI (kg/m^2^)	(*n* = 8)	(*n* = 27)
20–29	3	13
30–39	4	10
40+	1	4
Mean ± SD	31.41 ± 6.43	31.61 ± 7.11

^*∗∗∗∗*^ *p* < 0.0001.

**Table 2:** Patient characteristics of Bioserve samples (*n* valid cases = 80).

	Males (*n* = 20)	Females (*n* = 60)
Age (years)		
25–39	—	2
40–59	12	50
60+	8	8
Mean ± SD	57.1 ± 8.55	53.93 ± 7.28
Weight (kg)		
50–75	4	32
76–99	13	22
100+	3	6
Mean ± SD^*∗*^	85.20 ± 12.31	77.80 ± 16.43
Height (cm)		
125–159	1	26
160–169	9	28
170+	10	6
Mean ± SD^*∗∗∗∗*^	172.95 ± 11.14	159.32 ± 7.96
BMI (kg/m^2^)		
20–29	14	32
30–39	6	22
40+	—	6
Mean ± SD	28.50 ± 3.44	30.71 ± 6.32

^*∗∗∗∗*^ *p* < 0.0001.

^*∗*^ *p* < 0.05.

**Table 3:** Panel of fatty acids tested against anti-LCSFA IgG.

Fatty acid	Degree of saturation	Carbon chain length	Double bond position
Butyric acid	Saturated	6	N/A
Palmitic acid	Saturated	16	N/A
Stearic acid	Saturated	18	N/A
Lignoceric acid	Saturated	24	N/A
Palmitoleic acid	Monounsaturated	16:1	cis-Δ9
Oleic acid	Monounsaturated	18:1	cis-Δ9
Elaidic acid	Monounsaturated	19:1	trans-Δ9
Linoleic acid	Polyunsaturated	18:2	cis,cis-Δ9,Δ12
Arachidonic	Polyunsaturated	20:4	cis,cis,cis,cis-Δ5,Δ8,Δ11,Δ14
Docosahexaenoic acid	Polyunsaturated	22:6	cis,cis,cis,cis,cis,cis-Δ4,Δ7,Δ10,Δ13,Δ16,Δ19

**Table 4:** Study participant clinical characteristics with complete datasets at baseline and 3 months, *n* = 13. Values are presented as the mean ± SD.

Variables	Baseline	3 months	Mean difference	*p* value
Anti-LCSFAlmitic acid IgG (corrected OD^a^)	0.79 ± 0.40	0.17 ± 0.09	0.71 ± 0.44	0.001
IL-1*β* (pg/mL)	1.09 ± 0.46	0.98 ± 0.38	0.100 ± 0.44	0.442
Fasting glucose (mg/dL)	153.77 ± 65.12	143.62 ± 61.95	10.15 ± 19.36	0.050
HbA1c (%)	7.7 ± 2.2	6.8 ± 1.4	0.9 ± 1.2	0.005
HbA1c (mmol/mol)	61 ± 24	50 ± 15	11 ± 13	0.005
Insulin (pmol/L)	90.48 ± 74.88	99.30 ± 115.68	−8.88 ± 64.74	1.00
Cholesterol (mg/dL)	170.23 ± 33.51	158.54 ± 38.40	11.69 ± 31.60	0.184
HDL cholesterol (mg/dL)	45.00 ± 8.19	48.00 ± 7.916	−3.00 ± 4.43	0.049
LDL cholesterol (mg/dL)	98.62 ± 25.92	93.15 ± 25.35	5.46 ± 21.89	0.382
Cholesterol/HDL ratio (%)	3.86 ± 0.85	3.35 ± 0.67	0.50 ± 0.63	0.023
Triglycerides (mg/dL)	249.38 ± 174.09	191.92 ± 36.15	57.46 ± 98.02	0.075
TANITA^b^ fat mass (kg)	36.15 ± 12.40	33.98 ± 11.63	2.16 ± 2.45	0.013
TANITA^b^ fat percent (%)	42.25 ± 7.27	40.56 ± 7.43	1.66 ± 1.97	0.034
Scale weight (kg)	83.74 ± 16.10	82.17 ± 15.38	1.56 ± 3.20	0.100
Waist circumference (cm)	101.43 ± 9.73	99.89 ± 10.33	1.54 ± 5.36	0.196
Hip circumference (cm)	111.05 ± 13.91	111.94 ± 12.76	−0.89 ± 3.82	0.294
BMI (kg/m^2^)	32.33 ± 6.33	32.11 ± 5.54	0.22 ± 2.64	0.039
DXA^c^-trunk fat (kg)	16.60 ± 43.79	15.66 ± 41.20	0.94 ± 1.06	0.007
DXA^c^-trunk percent fat (%)	37.55 ± 6.78	36.36 ± 6.49	1.18 ± 1.68	0.041
DXA^c^-total fat (kg)	31.81 ± 11.06	30.27 ± 10.21	1.53 ± 1.86	0.013
DXA^c^-total percent fat (%)	36.94 ± 8.11	35.90 ± 7.85	1.04 ± 1.18	0.012

^a^Optical density.

^b^A brand of bioelectric impendence technology which measures body composition. TANITA scales measure within 5% of DXA.

^c^DXA: dual-energy X-ray absorptiometry.

**Table 5:** Anti-LCSFA antibodies and IL-1*β* from serum of Hispanics with type 2 diabetes correlate with body composition and HbA1c, respectively.

Variables	*N*	Spearman correlation	*p* value
Correlated with anti-LCSFA IgG			
DXA^a^-total fat (kg)	27	−0.429	0.025
DXA^a^-trunk percent fat (%)	27	−0.404	0.003
Correlated with IL-1*β*			
HbA1c (%)	27	0.383	0.048

^a^DXA: dual-energy X-ray absorptiometry.

## Abbreviations

PA: Palmitic acid
DXA: Dual X-ray absorptiometry
DMF: Dimethylformamide
ETOCOCL: Ethylchloroformate
OD: Optical density
SFA: Saturated fatty acids.
